# Hydroacoustic surveys reveal high sediment carbon accumulation in an urban lake

**DOI:** 10.1007/s11368-025-04029-3

**Published:** 2025-04-21

**Authors:** José R. Paranaíba, Quinten Struik, Melisa Rodriguez, Sebastian Sobek, Sarian Kosten

**Affiliations:** 1 Department of Ecology, Radboud Institute for Biological and Environmental Sciences, Radboud University, Nijmegen, The Netherlands; 2 Limnology, Department of Ecology and Genetics, Uppsala University, Uppsala, Sweden

**Keywords:** Sedimentation, Organic carbon, Inorganic carbon, Human-made lake

## Abstract

**Purpose:**

Insight into the distribution and sedimentation patterns of organic and inorganic carbon (OC and IC) in urban lake sediments is essential for understanding their role in the carbon (C) cycling of inland waters and supporting effective ecosystem management.

**Methods:**

This study investigated the spatial variability of sediment OC and IC accumulation in a mesotrophic human-made urban lake (Lake Berendonck; 45 ha) by combining high-resolution hydroacoustic sub-bottom profiling surveys and sediment coring.

**Results:**

The results revealed strong spatial variations in sediment C accumulation rates. Deep central and southeastern areas of the lake exhibited relatively high C deposition, even though deep areas with low C content were also found. Lake Berendonck had a mean ± standard deviation sediment accumulation rate of 0.7 ± 0.5 cm year^− 1^, with areal OC and IC accumulation rates ranging between 24 and 557 and 3–37 g m^− 2^ year^− 1^, respectively. Lake Berendonck’s mean sediment OC accumulation rate (155 g m^− 2^ year^− 1^) was approximately four times higher than the mean OC accumulation rate of global lakes (37 g m^− 2^ year^− 1^), while Lake Berendonck’s mean IC accumulation rate (12 g m^− 2^ year^− 1^) falls in the mid-range for global lakes and seas.

**Conclusions:**

Our findings indicate that C accumulation is highly variable in space and that spatially integrated data are needed to estimate C stocks and unravel within-lake C processes reliably. Furthermore, this study highlights that the OC accumulation in Lake Berendonck ranks among the highest rates observed in global lakes with similar surface areas (0.4–0.5 km^2^). This underscores the global importance of small urban water bodies in C cycling, particularly as key C storage systems.

**Supplementary Information:**

The online version contains supplementary material available at 10.1007/s11368-025-04029-3.

## Introduction

Urbanization is rapidly expanding worldwide, significantly impacting ecosystems, including freshwater lakes. As cities expand, their surrounding environments undergo substantial modifications, leading to alterations in land use patterns and nutrient cycling (Foley et al. 2005; Heathwaite 2010). Urban lakes, which provide important ecosystem services such as flood control, maintenance of biodiversity, and recreation, are particularly susceptible to the detrimental effects of anthropogenic activities (Pickard et al. 2021; Goeckner et al. 2022; Liu et al. 2022).

Frequently, urban lakes and other aquatic systems influenced by urbanization (e.g., reservoirs, ponds, rivers, and streams) receive elevated nutrient and organic matter loads through various pathways, including stormwater runoff, wastewater discharges, and atmospheric deposition (Bernhardt et al. 2008; Schwarzenbach et al. 2010; Wong et al. 2012). Excessive nutrient enrichment can lead to eutrophication, which is characterized by increased algal blooms, reduced water clarity, and bottom water oxygen depletion, thereby degrading system-wide water quality and biodiversity (Carpenter 2008; Smith and Schindler 2009; Moss et al. 2011). Eutrophication can also enhance carbon (C) accumulation through increased production of autochthonous organic matter driven by intense primary productivity. A portion of this C is exported to sediments, where it can be buried and sequestered over the long term (Holgerson et al. 2023). Therefore, C plays a critical role in the metabolism and productivity of freshwater ecosystems (Canfield et al. 2005; Battin et al. 2009). The distribution and accumulation of organic and inorganic forms of C in lake sediments reflects past and present environmental conditions, providing valuable insights into the historical nutrient dynamics and ecosystem status (Mendonça et al. 2014; Stockhecke et al. 2014; Maavara et al. 2015).

Both OC and IC (the latter mostly in the forms of carbon dioxide (CO_2_), carbon hydroxide (HCO_3_^−^), and calcium carbonate (CaCO_3_) enter a lake in several ways, such as through watershed runoff, groundwater infiltration, inflow of plant and animal material, atmospheric deposition, or by in situ primary production. While OC accumulation and burial in lake sediments has been shown to play a crucial role in C cycling in global inland waters (Downing 2010; Mendonça et al. 2017; Anderson et al. 2020), the accumulation of IC represents a largely understudied, but in many cases (e.g., hard water lakes), a significant portion of the C pool present in lake sediments (Finlay et al. 2010). The increased inputs of C into aquatic ecosystems can influence sedimentation rates, composition, and nutrient dynamics, potentially leading to alterations in the functioning of the ecosystem (Heathcote and Downing 2012; Anderson et al. 2014). Since sedimentation patterns can be very heterogeneous in space (Mendonça et al. 2014), understanding the spatial sedimentation of OC and IC in urban lakes is important for effective lake management and restoration efforts. While the importance of studying C accumulation and burial in lakes is widely recognized (Heathcote et al. 2015; Mendonça et al. 2017), the paucity of high-resolution spatial studies in urban lake environments is remarkable. Traditional sediment sampling approaches often rely on a limited number of sediment cores resulting in limited spatial coverage, which may not adequately capture whole-system spatial heterogeneity of sediment and anthropogenic impacts (Mendonça et al. 2014; Gallmetzer et al. 2016). High-resolution spatial mapping techniques, such as hydroacoustic sub-bottom profiling, provide the possibility to non-invasively investigate in detail sediment distribution patterns across different lake regions (Mendonça et al. 2014, 2016; Sotiri et al. 2021).

Thus, by combining sediment core analysis with hydroacoustic sub-bottom profiling in an urban lake in the Netherlands, our study aimed to assess the spatial variability of OC and IC accumulation, explore the influence of lake bed and surrounding terrestrial environments on sediment C distribution patterns, estimate system-wide sediment OC and IC stocks, and compare our OC and IC accumulation rates with those of global lakes and reservoirs.

## Methods

### Study area

**Fig. 1:**
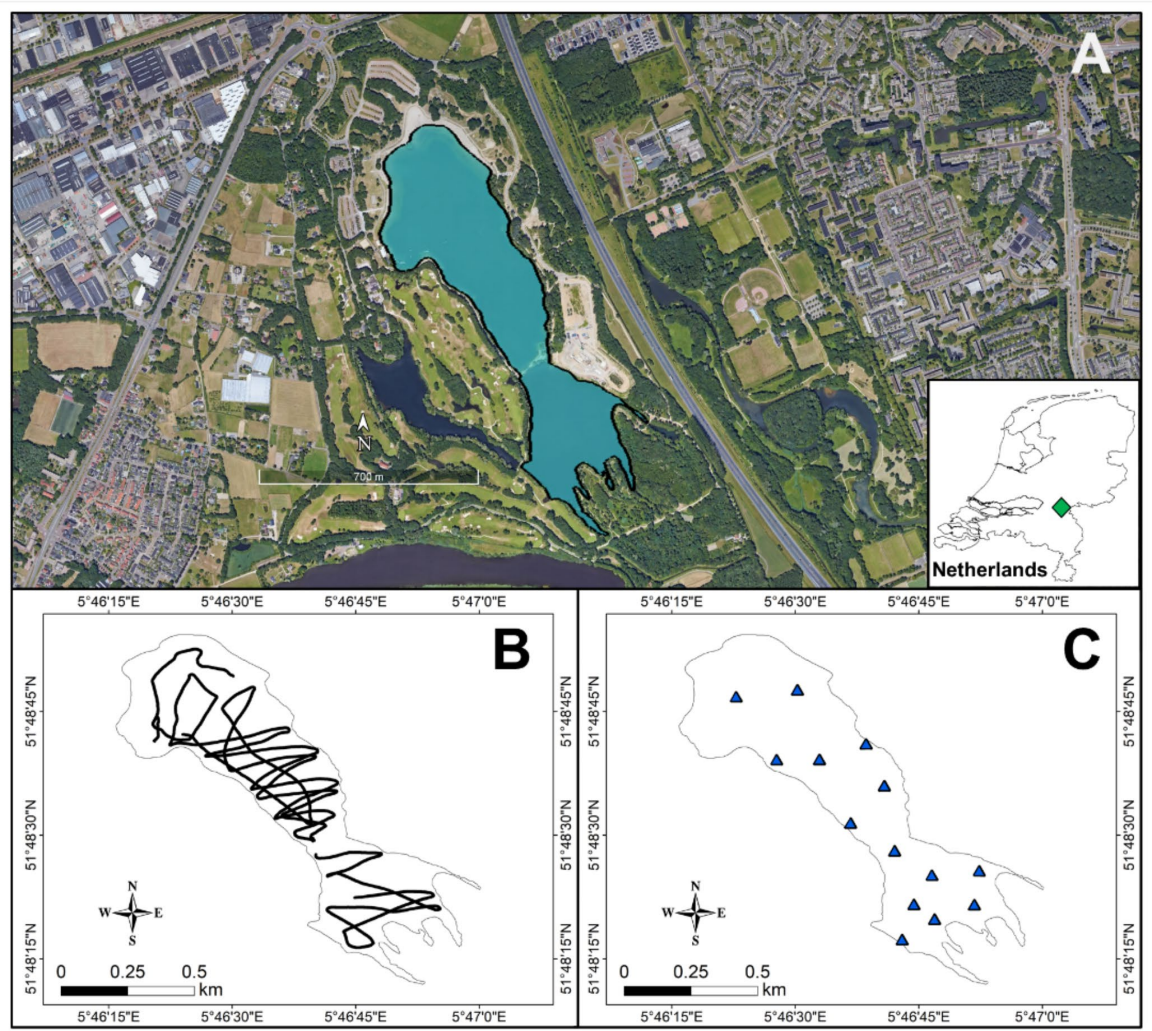
(**A**) Aerial image of Lake Berendonck (blue area) and its surroundings. (Source: Google Earth Pro, Imagery date: 20-08-2023). (**B**) Hydroacoustic sub-bottom profiling transects (black lines). (**C**) Locations where sediment cores were collected to measure sediment thickness and analyze the C content in different sediment depths (blue triangles)

Lake Berendonck (51⁰48.647’N, 5⁰46.576’E) is an urban lake located in Wijchen, in the southeast of the Netherlands (Fig. 1). The lake is part of a broader urbanized region known as the Arnhem-Nijmegen green-metropolitan area. The lake has an area of 0.45 km^2^, with an average depth of 11 m (maximum depth: 19 m), and was built in 1976 by excavating sand and gravel, materials which were used in the construction of the highway situated on the east side of the lake (Fig. 1). After the excavation, the site was transformed into a lake for recreational purposes. The lake serves as a popular destination for swimming, diving, fishing, sunbathing, boating, and other water-based activities, however, the contribution of these activities to the lake C cycle remains unknown. A wellness center is located in the southeast part of the lake, trees grow along the southern end (broadleaf trees, mainly European beech (*Fagus sylvatica*; 10–40 m tall) and English oak (*Quercus robur*; 10–25 m tall) (van der Meijen et al. 1983), a golf course can be found in the western part, and the northern portion locates the most suitable place for swimming and water activities (Fig. 1A). The lake is mainly rainwater-fed, but it also receives water through groundwater infiltration (van Hal and Lürling 2004). Runoff from the nearby golf course potentially is an important nutrient source along with atmospheric deposition and groundwater inflow originating from the Wijchens Ven and Hatertse Vennen moorland pools (Zaal 2003).

Lake Berendonck is classified as a mesotrophic lake (annual mean total phosphorus = 50 ± 35 µg L^− 1^, annual mean total nitrogen = 780 ± 30 µg L^− 1^, and annual mean chlorophyll-*a* = 5 ± 4 µg L^− 1^), with epilimnion concentrations being generally lower than in the hypolimnion (van Hal and Lürling 2004). The lake stratifies between May and October, with the thermocline and oxycline generally occurring at ~ 6 m, and bottom waters usually being sub-oxic (< 1 mg L^− 1^) throughout the summer (van Hal and Lürling 2004).

### Geospatial acoustic sediment profiling and analysis

**Fig. 2:**
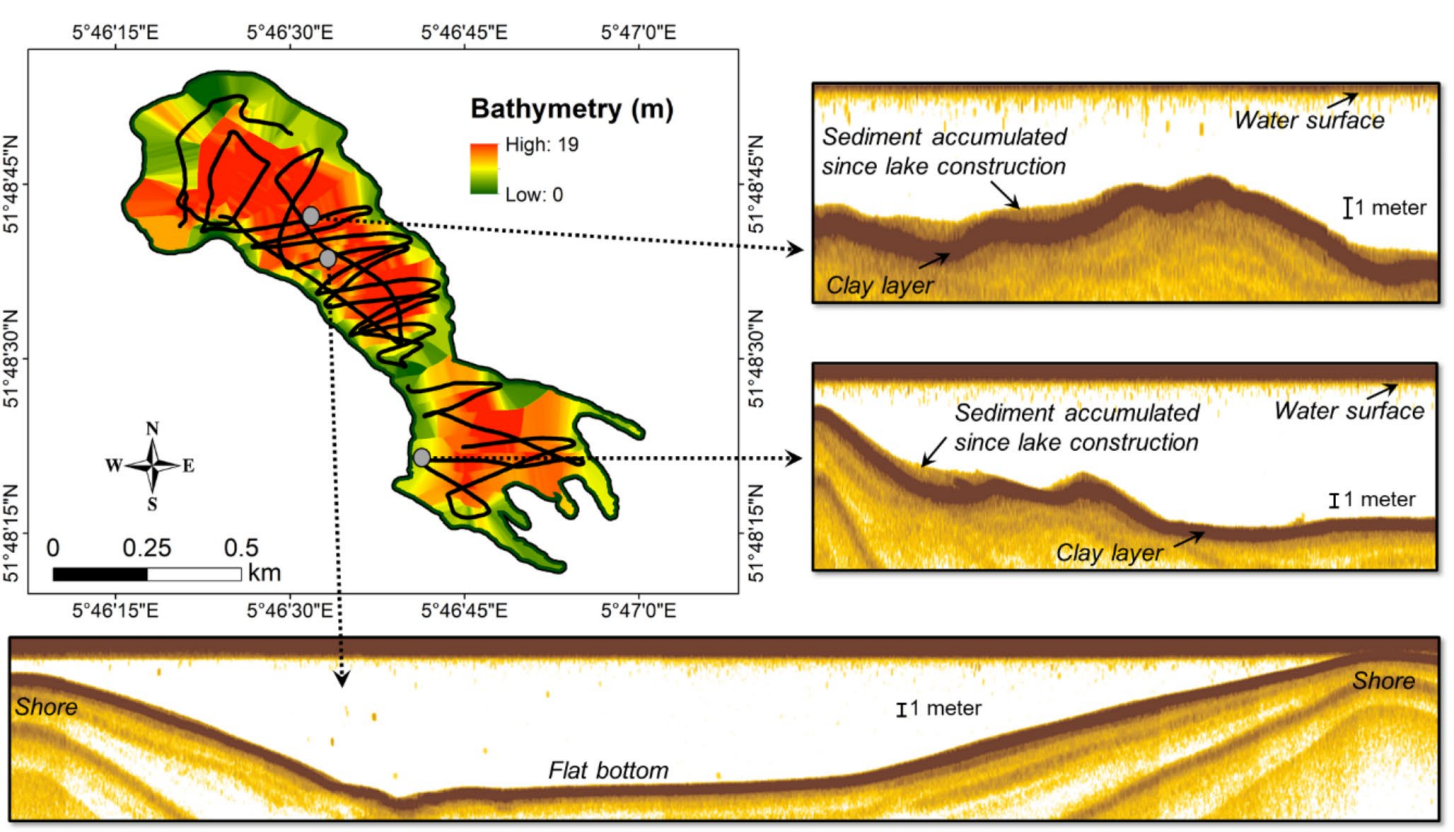
Different hydroacoustic sub-bottom profiling sections in Lake Berendonck. The dark brown layer represents the clay layer until which excavation took place when the lake was constructed. The beige layer on top of the clay represents the thickness of the sediment accumulated since the creation of the lake. The transect in the bottom part shows a hydroacoustic sub-bottom profiling transect from one shore to another. The map shows the bathymetry (m) of Lake Berendonck obtained during the hydroacoustic sub-bottom profiling survey in March 2023. Red regions represent deep locations, whereas green regions represent shallow locations

In March 2023, we performed shore-to-shore transects (Fig. 1B) with a multi-frequency single-beam hydroacoustic sub-bottom profiler mounted with an acoustic source/receiver (range of soundwaves used: 4–15 kHz; ping rate up to 40 pings s^− 1^; beam angle: 1–2 degrees; Innomar SES-2000) in order to determine sediment thickness and sedimentation patterns in the lake bottom since its creation (Mendonça et al. 2014). The boat speed was set at 3 km h^− 1^. Sound waves were continuously sent through the water column until the bottom of the lake and penetrated the soft sediment but hardly penetrated the denser pre-flooded soil, in our case composed of clay (Fig. 2). This enabled us to identify the lake bottom and later to measure the sediment layer accumulated since the construction of the lake and determine whole-lake sediment volume at a high spatial resolution (Fig. 2), similar to Mendonça et al. (2014). Real-time geographic positioning data were concomitantly recorded with a portable GPS device (Garmin GPS 19x HVS– NMEA 0183) coupled to a computer.

Hydroacoustic transects (Fig. 2) were processed through the software ISE 2.9.68 (Innomar) using an acoustic wave propagation between 1400 and 1600 m s^− 1^, which is within the typical range for muddy sediments. Thus, sediment thickness was determined by logging the current lake bottom (i.e., sediment surface) and the clay surface (i.e., sub-bottom) at a depth resolution of 5 cm, with the sediment thickness being the distance between the surface of the sediment and the surface of underlying clay. Sediment cores (*see below*) were collected in different regions of the lake in order to validate the acoustically derived sediment thickness.

### Sediment coring and analysis

In total, 14 sediment cores (6 cm inner diameter) were collected throughout the lake (Fig. 1C) using a gravity corer equipped with a hammer device (UWITEC– Mondsee, Austria), of which, three cores were used only to validate the acoustically-derived sediment thickness observed in the hydroacoustic survey, and 11 cores were also used for laboratory analysis. Cores were hammered into the sediment to ensure that the bottom clay layer was reached so that the entire lake sediment layer could be recovered. The clay layer was reached in all sediment cores sampled (ocular inspection). The sediment cores were sealed with plastic lids and immediately transported to the laboratory for slicing and subsequent laboratory analysis. In the laboratory, sediment cores were sliced in samples of 2 cm thick, and were stored in sealed plastic bags at -20 ºC until further analysis.

For each sediment slice, water content was determined by drying the sample at 70 ºC for 72 h. After this procedure, the dry sample was homogenized using a pestle and a mortar and subdivided for analyses of organic matter, IC, and total C. For organic matter analysis, 20 g of dry sub-samples were combusted at 500 ºC for 12 h (Wang et al. 2011), and the organic matter content was estimated as the difference between the dry weight and the weight of the sample after loss of ignition (LOI) at 500 ºC. Subsequently, the sediment IC fraction of each slice (derived from the burning of CaCO_3_) was estimated by the LOI at 800 ºC for another 12 h (Wang et al. 2011). To estimate the total carbon content in each sediment slice, 40 mg of dry sub-samples were analyzed using an elemental CNS analyzer (Vario MICRO cube; Elementar Analysensysteme, Langenselbold, Germany) (Vroom et al. 2020). Accordingly, the OC content present in each sediment slice was estimated by the difference between the total C content obtained by the CN analysis and the IC content obtained by the LOI method.

### Data analysis

Organic and inorganic C masses (g; OC and IC) were expressed per g dry weight of sediment. Total sediment core OC and IC contents were calculated by the sum of C masses in all 2 cm sediment slices. Areal OC and IC accumulation rates (g OC and IC m^− 2^ year^− 1^) from each sediment core were estimated from the total C masses (g C), core surface area (0.0028 m^2^), and the lake age (47 years in 2023). The sediment accumulation rate (SAR; cm year^− 1^) was estimated from sub-bottom profiling and the time since the creation of the lake. Given that Berendonck’s SAR positively correlated with OC and IC accumulation rates in all coring sites (see Sect. 3 below), we used the regression models between sub-bottom-derived SAR and OC and IC sedimentation rates to estimate the spatially resolved OC and IC sedimentation rates in Lake Berendonck. Accordingly, the total lake sediment stocks of OC and IC (in tons) were estimated as the product of the whole-lake OC and IC sediment contents, lake area, and age. Bathymetric data and SAR were interpolated to the whole-lake surface using the Inverse Distance Weighting (IDW) algorithm in ArcGIS (version 10.8, ESRI), resulting in a grid of 246 columns and 236 rows.

## Results

### Hydroacoustic sub-bottom profiling survey

**Fig. 3:**
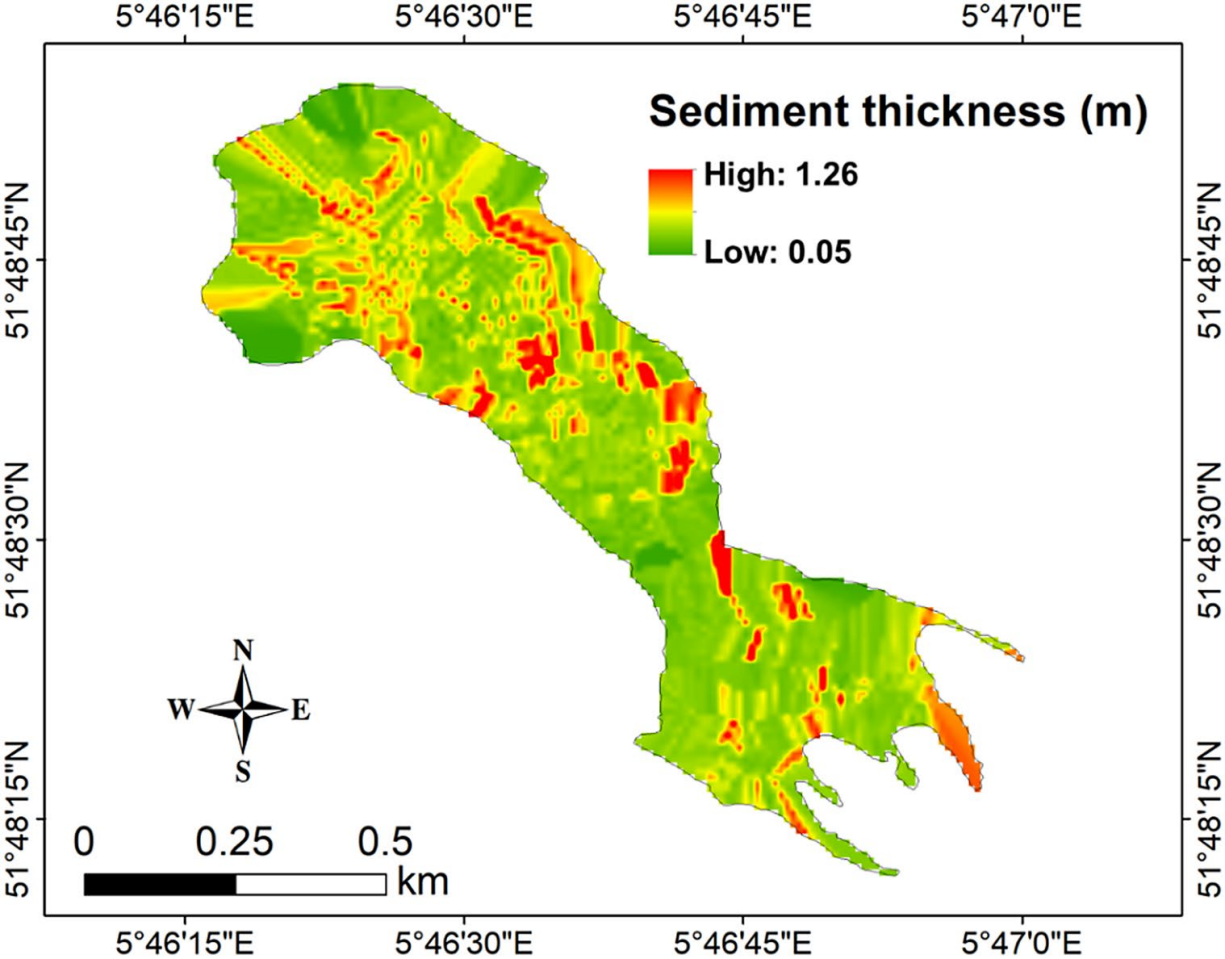
Spatial distribution of Berendonck’s sediment thickness (m) derived from hydroacoustic sub-bottom profiling survey in March 2023. Green regions indicate thin layers of sediment (implying low sedimentation rates), whereas red regions indicate thicker sediment layers (implying high sedimentation rates)

Sub-bottom profiling surveys showed a strong spatial variability of sediment accumulation in Lake Berendonck (Fig. 3). The sometimes uneven sub-bottom topography led to patchy sediment accumulation, whereas more uniform sedimentation occurred in flatter areas (Fig. 2). Sediment thickness varied from 0.05 to 1.26 m (mean: 0.36 m; Fig. 3), resulting in a mean SAR of 0.7 cm year^− 1^ (range: 0.1–2.6 cm year^− 1^) since Lake Berendonck’s creation. Overall, high SARs were more common in the eastern and southern parts of the lake (Fig. 3). Although deep regions generally showed high sedimentation rates, some deep regions with low sediment accumulation were also found (Figs. 2 and 3). In general, little sediment accumulation (0–0.1 cm year^− 1^) occurred along the margins, but on the gentle slopes towards the central part of the lake, relatively thick sediment layers were present (0.7–1 m; Fig. 3). The total amount of sediment accumulated in Lake Berendonck since its creation (i.e., 1976) is estimated to be ~ 1.5 × 10^5^ m^3^.

### Sediment core profiles and areal OC and IC sediment content

**Fig. 4:**
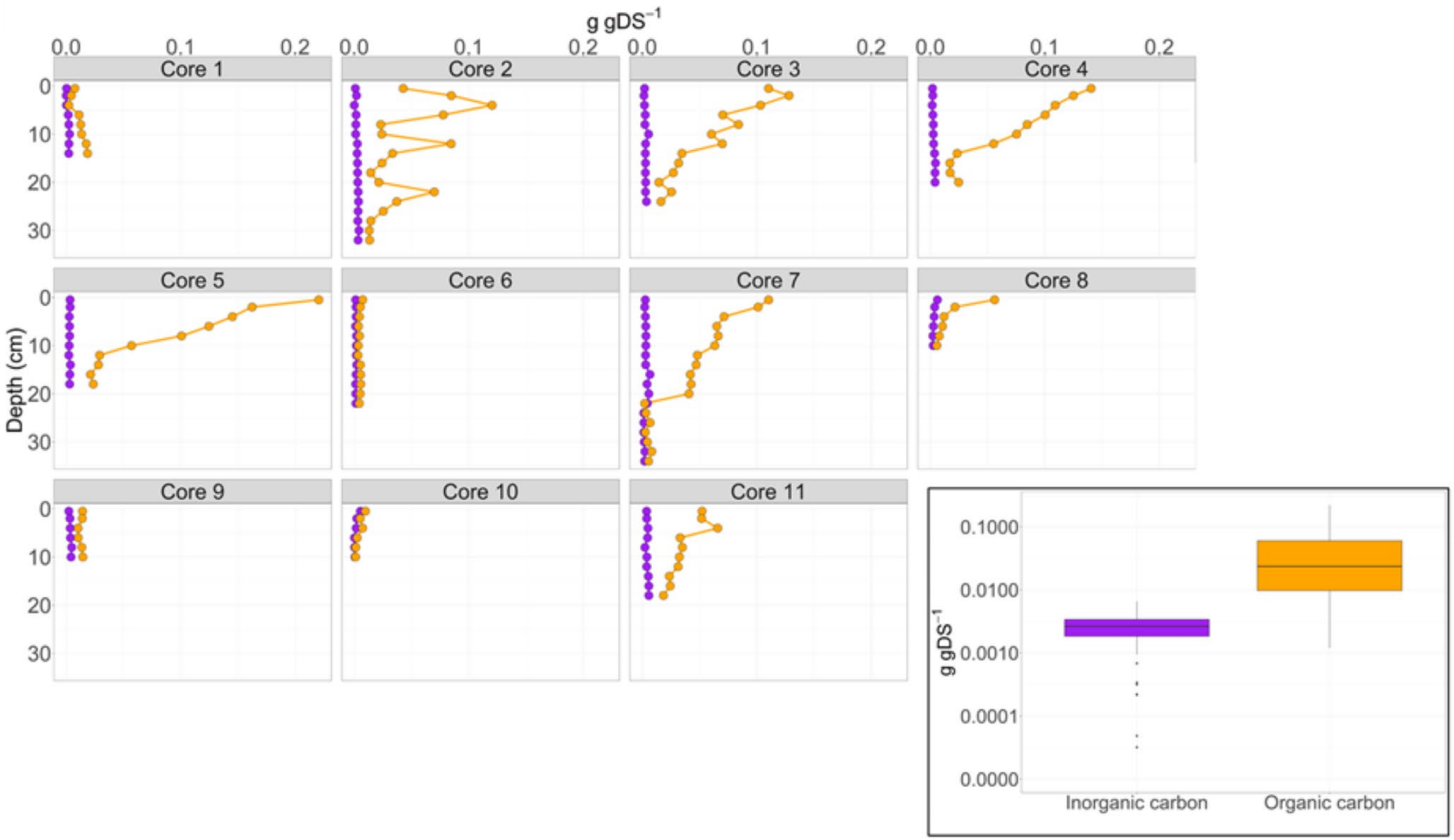
Organic carbon (OC; orange) and inorganic carbon (IC; purple) contents (gram per gram of dry sediment: g gDS^− 1^) in different layers of sediment cores collected in Lake Berendonck in March 2023. The boxplots show the range of all sediment OC and IC contents (axis shown in logarithmic scale). Boxes indicate 25–75th percentiles, whiskers show the 5–95th percentiles, and the horizontal lines represent the median

The sediment cores collected at 11 locations in Lake Berendonck showed remarkable variability in OC content, both between sites and between different sediment layers (Figs. 4 and S1). The amount of OC and IC accumulated per square meter in the sediment of Lake Berendonck, hereafter referred to as areal OC and IC accumulation rates, ranged from 24 to 557 g m^− 2^ year^− 1^ (mean ± SD: 155 ± 111 g m^− 2^ year^− 1^) for OC, from 3.5 to 37 g m^− 2^ year^− 1^ (mean ± SD: 12 ± 7 g m^− 2^ year^− 1^) for IC. Accordingly, we estimated the total sediment OC and IC stocks accumulated since Berendonck’s creation to be ~ 3.3 Gg and ~ 0.26 Gg, respectively.

## Discussion

### Patterns in SAR, OC, and IC accumulation in lake berendonck

Sediment accumulation rates and, hence, sediment OC and IC contents were higher in the southeast parts of the lake (Figs. 3 and S1)– regions surrounded by trees and the wellness center (Fig. 1). Wind direction may play an important role in regulating SAR and C sedimentation patterns in Lake Berendonck. Prevailing westerly winds (KNMI 2022) generate currents that carry suspended particulate organic matter toward the east, where they can settle. In addition, these winds can favor the movement of sediments and other particles suspended at the bottom of the lake in opposite directions (due to mass circulation), which may favor the observed high spatial variability of SAR. Furthermore, events of cyanobacterial blooms are recurrent in Lake Berendonck between spring/summer time (van Hal and Lürling 2004), which, allied to the relatively small size of the lake, may also be contributing to the high areal OC sedimentation rate estimated in our study (Holgerson et al. 2023). The prevalence of trees in the southeast region may also be amplifying the lateral flow of terrestrial C into the lake. Drainage pipes can be found on the east side of the lake, which drain excess water and matter (containing C and nutrients) from the adjacent land into the lake. Reed banks (such as *Phragmites australis*) are present right after these drainage pipes, to slow down water flow, which in turn contributes to the enhanced deposition of sediment (and consequently C) in these regions (Fig. 3). In addition, the detritus of these plants might contribute to C sedimentation locally.

Although in deeper areas generally a high SAR is expected, our findings reveal that both elevated and low SAR (Fig. 3) occur in deep regions (Figs. 2 and 3). This variability may be attributed to factors such as local hydrodynamics and the nature of the lakebed (Mendonça et al. 2014). The irregular sub-bottom morphology (Fig. 2) resulted in patchy SAR (Fig. 3), even though some flatter bottom areas also showed more uniform sedimentation (Fig. 2). Consequently, sediment coring data alone would have provided only limited characterization of sedimentation processes within the system. In addition, extrapolation of SAR obtained from a core in the deeper part of a lake to the entire lake area may lead to a considerable over or underestimation of the lake C stock. Therefore, deploying techniques that generate spatially resolved maps of lakebed morphology and sediment thickness (such as hydroacoustic profilers) contributes to more accurate quantification of C stocks and sediment-related processes within lakes.

### Berendonck’s C pools in comparison to other aquatic systems and temperate forests estimates

**Fig. 5:**
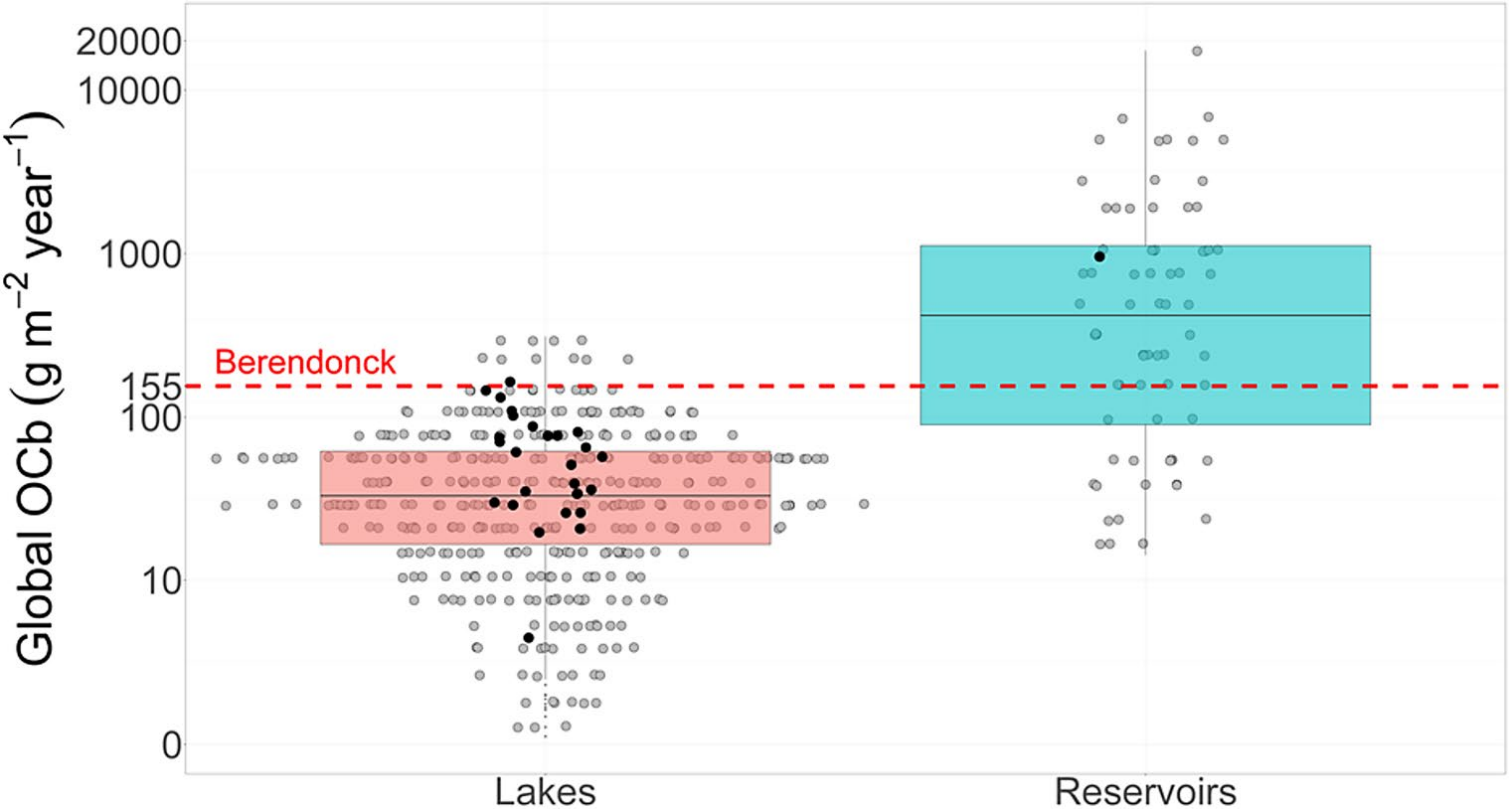
Global organic carbon burial (OCb; g m^− 2^ year^− 1^) rates in lakes and reservoirs available in Mendonça et al. (2017). The red dashed line indicates Berendonck’s OC accumulation rate estimated in this study (155 g m^− 2^ year^− 1^). Note that the y-axis is shown in pseudo-log_10_ scale. Black dots represent systems (lakes and reservoirs) with surface areas similar to Lake Berendonck (Berendonck surface area: 0.45 km^2^; selected system areas: 0.4–0.5 km^2^)

Our refined spatial analysis of lake sediment revealed that the relatively small Lake Berendonck (0.45 km^2^) exhibits a high OC accumulation rate (155 g m^− 2^ year^− 1^) compared to the global lake average (37 g m^− 2^ year^− 1^; 344 observations), while its rate falls in the lower range for reservoirs worldwide (average 165 g m^− 2^ year^− 1^; 59 observations) (Fig. 5). Narrowing down the comparison to lakes of similar surface area (0.4–0.5 km^2^), we find that the OC accumulation rate in Lake Berendonck falls at the higher end of the range observed (Fig. 5). The average IC accumulation rate found in Lake Berendonck, on the other hand, is situated in the middle range of the observations available in the literature for lakes and seas around the globe (Table S1).

Comparing the C accumulation rate of Lake Berendonck (~ 170 g m^− 2^ year^− 1^) to the C sequestration capacity of temperate forests (~ 230 g m^− 2^ year^− 1^; corrected for C losses via dissolved OC and IC export) (Arets 2018) indicates that these rates are comparable in order of magnitude. This highlights the potential of lakes as significant C sinks. However, unlike forests, C accumulation and burial in lakes are not currently recognized in C credit markets. Integrating this potential into the design and management of aquatic ecosystems, particularly urban ones, could unlock a novel avenue for C mitigation strategies. However, it is key to consider the fate of the settled C. While sedimentation removes C from short-term circulation, some may eventually be re-mineralized and released back into the water column and subsequently into the atmosphere as the greenhouse gases CO_2_ and methane (CH_4_), potentially turning lakes into sources of CO_2_-equivalents to the atmosphere despite high sediment C accumulation (Sobek et al. 2009; Mendonça et al. 2016; Grasset et al. 2020).

## Conclusions

Here we showed for the first time in an urban lake that the use of high-resolution hydroacoustic sub-bottom profiling was effective in quantifying the spatial variation of C sedimentation, including the identification of ‘hotspots’ for sediment accumulation (Figs. 3 and S1). By considering finely detailed spatial variations in C sedimentation, water managers can address lake-related challenges (e.g., internal nutrient loading, silting) with greater precision. The use of the hydroacoustic approach enables a more targeted allocation of efforts and resources when implementing specific management strategies (such as dredging; Nürnberg 1991; Lürling and Mucci 2020), thereby enhancing its overall effectiveness and potentially decreasing its costs.

In many situations, lake management aims to prevent or mitigate phytoplankton blooms, which disrupt ecosystems and harm water quality (Smith and Schindler 2009). Yet, in relatively deep lakes (as in the case of Lake Berendonck), a twist emerges: phytoplankton blooms can also boost atmospheric C sequestration (Anderson et al. 2014). When phytoplankton dies, it sinks to the lake bottom, accumulating OC in the sediments. However, this process carries risks. Phytoplankton blooms worsen eutrophication, depleting oxygen and releasing nutrients from the sediment, which fuel further phytoplankton growth. Additionally, the decomposition of phytoplankton-based organic matter in low-oxygen conditions produces CH_4_ out of CO_2_-derived C (Bastviken 2009). So, while promoting phytoplankton blooms may increase C accumulation, simultaneously it may stimulate CH_4_ emissions (Grasset et al. 2020), offsetting lakes’ C-sequestration potential. Navigating these trade-offs requires a nuanced understanding of lake dynamics and careful management strategies to achieve an optimal level of productivity in lakes, where increased CO_2_ sequestration occurs without triggering CH_4_ emissions. It underscores the importance of interdisciplinary research and adaptive management approaches to address the multifaceted environmental issues facing freshwater ecosystems.

## Electronic supplementary material

Below is the link to the electronic supplementary material.

Supplementary Material 1

## Data Availability

Data and metadata used in this study are available at: 10.17026/LS/LPUJ5U.
