# Supplementary material for: Hydroacoustic surveys reveal high sediment carbon accumulation in an urban lake
Source: J Soils Sediments. 2025 Apr 21;25(5):1713–21. doi: 10.1007/s11368-025-04029-3 (PMC12065750; doi:10.1007/s11368-025-04029-3)
Supplement: Supplementary file 1 — Supplementary Material 1 [file 11368_2025_4029_MOESM1_ESM.pdf]

Supplementary information for:

Hydroacoustic surveys reveal high sediment carbon accumulation in an urban lake

José R. Paranaíba[1,*], Quinten Struik[1], Melisa Rodriguez[1], Sebastian Sobek[2], Sarian Kosten[1]

[1] – Department of Ecology, Radboud Institute for Biological and Environmental Sciences, Radboud University, Nijmegen, The Netherlands

[2] – Limnology, Department of Ecology and Genetics, Uppsala University, Uppsala, Sweden

Corresponding author:

* jose.paranaiba@ru.nl

Heyendaalseweg 135, 6525 AJ – Nijmegen, Netherlands

This supplementary document contains 3 pages, 1 table, and 1 figure.

Supplementary tables:

Table S1: Comparison of Berendonck's mean inorganic carbon accumulation rate (IC; g m^{-2} $year^{-1}$) with data available in the literature for lakes and seas around the globe. Plus/minus variation represents the standard deviation.

System	IC accumulation rate (g m^{-2} $year^{-1}$)	Reference
**Berendonck**	12.2 ± 7	***This study***
**Lake Gollinsee**	7	Brothers et al. (2013)
**Lake Van**	5	Einsele et al. (2001)
**Lake Chad**	2.4	Einsele et al. (2001)
**Great salt lake**	10.2	Einsele et al. (2001)
**Caspian sea**	34.3	Einsele et al. (2001)
**Lake Titicaca**	16.2	Einsele et al. (2001)
**Lake Constance**	37.8	Einsele et al. (2001)
**Lake Tanganyika**	0.54	Einsele et al. (2001)
**Lake Victoria**	0.12	Einsele et al. (2001)
**Lake Malawi**	0.84	Einsele et al. (2001)
**Black sea**	27.6	Einsele et al. (2001)
**Lake Qinghai**	11 ± 4.5	Chen et al. (2021)

Supplementary figures:

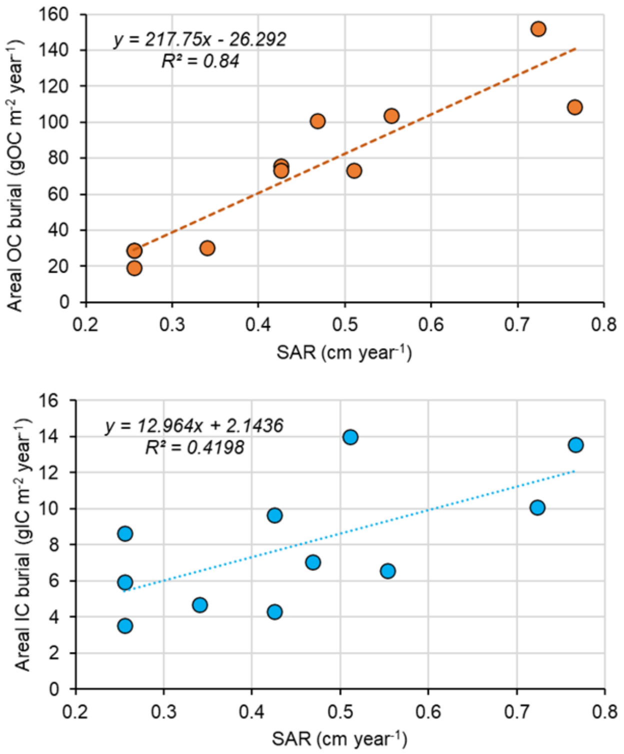

Figure S1: Relationship of areal organic carbon (OC; orange) and inorganic carbon (IC; blue) accumulation rates with sediment accumulation rate (SAR) of Lake Berendonck.
